# A Method for Computerized Olfactory Assessment and Training Outside of Laboratory or Clinical Settings

**DOI:** 10.1177/20416695211023953

**Published:** 2021-06-11

**Authors:** Simon Niedenthal, Johannes Nilsson, Teodor Jernsäther, David Cuartielles, Maria Larsson, Jonas K. Olofsson

**Affiliations:** School of Arts and Communication, Malmö University, Malmö, Sweden; Department of Psychology, Stockholm University, Stockholm, Sweden; School of Arts and Communication, Malmö University, Malmö, Sweden; Department of Psychology, Stockholm University, Stockholm, Sweden

**Keywords:** smell training, olfactory assessment, olfactory displays, olfactory interactions

## Abstract

There are currently few ways to reliably and objectively assess olfaction outside of the research laboratory or clinic. The COVID-19 pandemic has highlighted the need for remote olfactory assessment; in particular, smell training at home is a promising method for olfactory rehabilitation, but further methodological advances might enhance its effectiveness and range of use. Here, we present Exerscent, a portable, low-cost olfactory display designed primarily for uses outside of the laboratory and that can be operated with a personal computer. Exerscent includes Radio Frequency Identification (RFID) tags that are attached to odor stimuli and read with a MFRC522 module RFID reader/antenna that encodes the odor in order to provide adaptive challenges for the user (e.g., an odor identification task). Hardware parts are commercially available or 3D printed. Instructions and code for building the Exerscent are freely available online (https://osf.io/kwftm/). As a proof of concept, we present a case study in which a participant trained daily to identify 54 odors, improving from 81% to 96% accuracy over 16 consecutive days. In addition, results from a laboratory experiment with 11 volunteers indicated a very high level of perceived usability and engagement. Exerscent may be used for olfactory skills development (e.g., perfumery, enology), and rehabilitation purposes (e.g., postviral olfactory loss), but it also allows for other forms of technological interactions such as olfactory-based recreational interactions.

There is a need for a wider range of olfactory assessments outside of research laboratories and clinics. Objective tests of olfactory performance constitute the gold standard of olfactory assessment. The most common tests involve multiple-choice responses that are easy to score and compare to a norm population; thus, they are suitable for validating olfactory dysfunctions and differentiating true impairments from malingering in the clinic (Doty et al., 1984; Oleszkiewicz et al., 2019). These tests are also used widely in basic and clinical olfactory research (Devanand et al., 2020; Olofsson et al., 2016). The downside of these tests is that they have a limited range of use; they either need to be administered by an experimenter, or, in the case of the Smell Identification Test that consists of a scratch-and-sniff booklet with multiple-choice odor identification, allowing for testing at home, they are discarded after use (Doty et al., 1984). The common olfactory assessment methods are thus not suitable for repeated and unsupervised assessment outside of a clinical or research setting. For this reason, subjective olfactory assessments provide a complementary source of information in situations where objective smell assessments are unavailable or impractical. Asking questions about subjective olfactory abilities requires no physical contact between participants and researchers or clinicians and can be conducted over the internet or via telephone. Subjective olfaction has been imperative in establishing smell loss as a primary symptom of COVID-19 (Parma et al., 2020). Furthermore, self-assessed smell loss predicts later dementia and mortality among elderly persons (Ekström et al., 2017; Stanciu et al., 2014). Despite these promising results, the subjective nature of these assessments and the weak association typically found between subjective and objective assessments led researchers to regard subjective assessments as less useful (Hannum et al., 2020; Marchese-Ragona et al., 2020; Welge-Luessen et al., 2005; White & Kurtz, 2003).

The COVID-19 pandemic has exacerbated the need for objective assessment of olfaction without personal contact between patient and physician or researcher. A recent study used an online application to collect intensity ratings of common household odors in order to monitor the recovery from COVID-19 (Iravani et al., 2020). This method enables repeated and remote olfactory intensity assessment but nevertheless relies on subjective reports. A prioritized goal is to advance olfactory assessment methodology to the point where we can conduct flexible and reliable measurement of objective rather than subjective, olfactory abilities in the homes of the patient or participant. Such methods would be useful both for assessment and diagnoses but also for smell training in the recovery phase. Prior studies have shown that smell training after a postviral olfactory loss leads to improved olfactory ability (Hummel et al., 2009; Sorokowska et al., 2017). Smell training has been recommended as a means to recover olfaction following COVID-19 (Hopkins et al., 2020). Smell training has also shown to enhance olfactory perception and cognition, and arguably even nonolfactory cognitive abilities, suggesting a wide range of applications (Birte-Antina et al., 2018; Olofsson et al., 2020). However, long-term training regimes in the home may confer issues with user compliance, transparency, and efficiency. In studies that employ exposure to physical diagnostic tools such as *Sniffin’ Sticks*, compliance is reinforced through regular phone reminders (Hummel et al., 2009), attractively designed materials (Birte-Antina et al., 2018), and is ultimately confirmed through retrospective reviews of “smell diaries” kept by participants (Birte-Antina et al., 2018, p. 218). Besides these measures, however, there are few possibilities to monitor participant activity and performance during the period of olfactory training. Uncertainty in compliance remains a well-recognized limitation in studies conducted outside of the lab with physical materials (Oleszkiewicz et al., 2018). Dropout and lack of compliance affects the validity of results obtained in randomized controlled trials, and potential clinical benefits are lost if participants do not fulfill the assigned training.

Portable digital olfactory displays may help overcome the current limitations in olfactory assessment. Barfield and Danas (1996) define an olfactory display “as a collection of hardware, software, and chemicals that can be used to present olfactory information” (p. 110). Digital artifacts offer obvious benefits when compared to standard physical tools such as odor flasks, pens, or scratch-and-sniff cards, including the ability to collect precise behavioral responses with a digital interface, present immediate feedback to the participant, use response speed or accuracy to create adaptive challenges, and to log and communicate user behavior. These features might be harnessed to increase compliance and engagement with training tasks, improve interventions, and facilitate better outcomes.

An olfactory display designed for regular, unsupervised training in the home over weeks or months must be simple, robust, and built upon principles of good interaction design. It should be *efficient, easy to learn,* and *enjoyable to use* (Sharp et al., 2007). Olfactory displays serve the roles of vaporizing scent materials and delivering them to the nose (Nakamoto, 2012). Many olfactory displays used in research involve pneumatics, air being pumped through odorized chambers and released by the nose of the participant. This feature is necessary when exact stimulus control is needed, but most olfactory assessments do not require such exact control.

Digital olfactory interactions may provide an increased opportunity for entertainment and voluntary engagement with odor applications. Digital games have been used as experiment stimuli within psychology research since the mid-1970s (Washburn, 2003). It is believed that games provide engaging, motivating, challenging, and familiar interactions that may be more enjoyable than standard psychology tests. Digital games might enable experimental control and data logging over repeated assessments (Calvillo-Gámez et al., 2011; Järvelä et al., 2012; Washburn, 2003). Olfactory games are not widely used in olfactory research, with a few exceptions. A recently published study used an olfactory game for perceptual and memory training, with relatively high levels of motivation and enjoyment throughout a 40-day training period (Olofsson et al., 2020). Although that study did not use a digital interface, it serves as an example of games as a mode of interaction, and how the range of interactions can be expanded further by means of digital olfactory displays.

## Technical Description

Our present work involves a hybrid digital/physical platform for conducting olfactory research. In our design, the user grasps small vials of odor material and moves them directly to the nose to sniff. A hardware box, plugged into a personal computer, registers the identity of the odor and interacts with software to produce response options and other feedback to the user on the computer screen. This design engages the simplest and most efficient means of an olfactory display: natural diffusion for vaporization and scent delivery through intuitive and familiar gestures of the user. It thus aims to combine efficiency and ease of use with the benefits of a digital, interactive device—including the ability to monitor subject performance in real time, as well as logging and archiving experimental data. We employ Radio Frequency Identification (RFID) tags as a means of linking individual scent units to an interface and database. These tags can be attached to any set of scent vials, in any number. RFID tags are a key tool for associating data with physical objects and materials, making them well suited for use in olfactory displays that incorporate liquid or solid scent containers. In recent years, RFID tags have been employed as part of the growing emphasis upon embedding data in objects that take part in larger networks: the “Internet of Things.” Passive RFID tags are a flexible means of embedding data: They are low-cost, long-lasting, and draw power from adjacent tag readers (Greengard, 2015).

**Figure 1.**
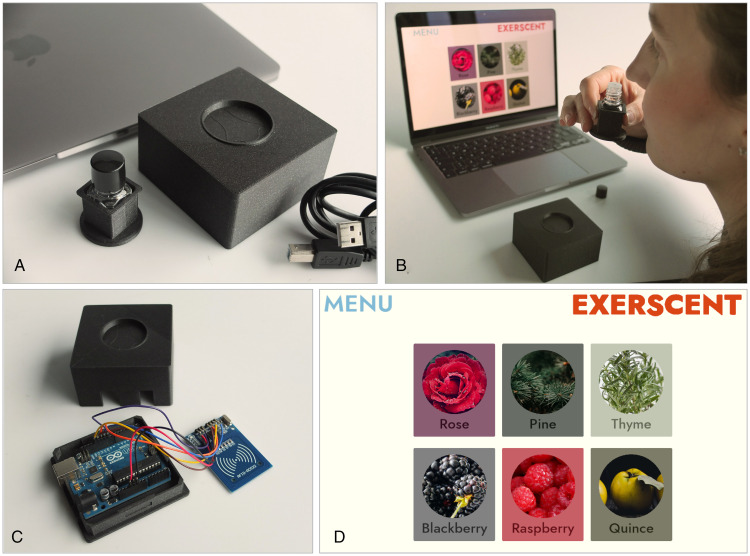
(a) Laptop, tagged scent material, MFRC522 module RFID reader/antenna, (b) smell training with laptop, (c) RFID tag reader and microcontroller, and (d) Exerscent interface.

This current platform consists of two main parts:

1. The **hardware** that is attached to the user’s desktop. We have chosen cost-effective, off-the-shelf components that offer reliability in nonlaboratory settings.
2. The game **software**, running on a personal computer (Figure 1).

## Hardware Solution

We use Mifare RFID tags operating at 13.56 MHz (within 2.5 – 3.3 V) which are read by an MFRC522 module RFID reader/antenna. The 13.56 MHz radio band is license free (ISM type B) and can be used freely by anyone. The RFID tags hold a string of data that is read by the RFID reader. Each tag has an individual string that corresponds to the smell of the vial to which it is attached, for example, “apple” and “lilac.” The data on the tags serve as identifier of the smells in the game software. To set up the RFID-tags, one can either write the data to the tags manually one by one by running code on the microprocessor, or one can have an external service write data to an array of tags.

The RFID tags are attached to the smell vials with adhesives. In the case of our pilot case study (see later), we have used scent materials from the *Le Nez Du Vin* (https://www.lenez.com/en/kits/wine/masterkit_54) wine aroma set that is commercially available. We have used a 3D printer to fabricate containers in which to place the smell vials. The containers serve three functions: to cover the preprinted identity number on the vials, to hide color variations of the liquids, and to create a surface for the adhesive RFID tags. The RFID reader and antenna module are fixed in a 3D-printed casing together with an Arduino UNO microcontroller that communicates between the tag reader physical interface and the digital graphical user interface. All Exerscent equipment is 3D printed out of polylactic acid and can be reproduced by using code found on the project website (https://osf.io/kwftm/).

## Software for Olfactory Assessment

When the user places the scent vial on the tag reader, a game engine is used to present different visual alternatives to the user through the graphical interface. The interactive environment is built in Unity Technologies’ game engine Unity3D and can be run on OSX and Windows operating systems. Programming in Unity3D is done in C#.

**Figure 2.**
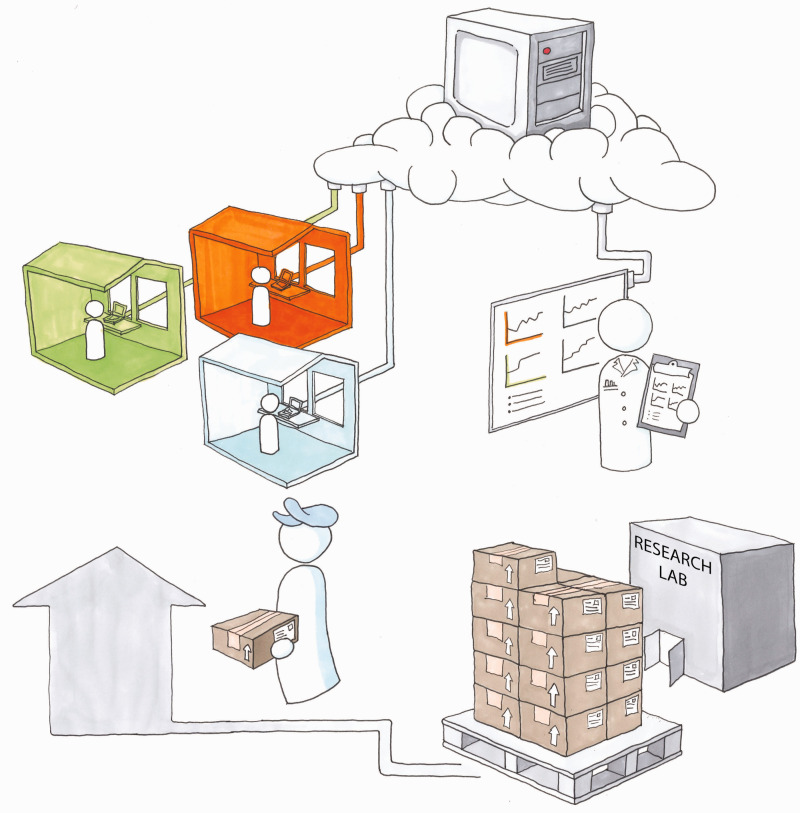
A framework for remote research assessment of olfactory abilities. Hardware is distributed to users, who conduct olfactory assessments at home via a computer interface that assembles user data for research or clinical use.

Hardware may be distributed to users for at-home use (Figure 2). The user logs in with a unique login code. From there, all interaction statistics are saved to an online Firebase database. No personal identification is collected. The user can login from any computer with internet connection, and researchers can access the data from anywhere.

## Computerized Assessment of Odor Identification

**Figure 3.**
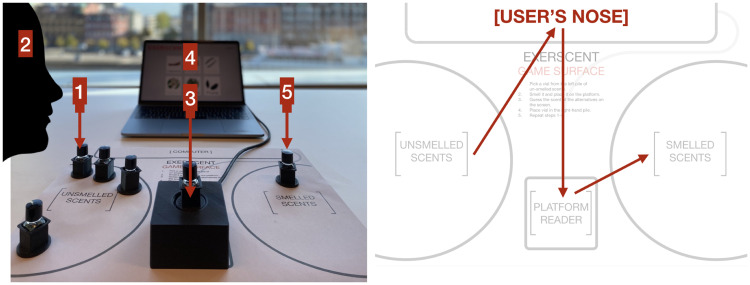
Interaction flow and board. Users move scents from left to right on the board, choosing from among unsniffed scents on the left (1), sniffing (2), placing scent on the platform reader (3), making a choice on the screen (4), and placing the sniffed scents to the right (5).

The user sniffs a vial, places the vial on the platform reader, and identifies the correct label corresponding to the scent among several alternatives presented on the computer screen (Figure 3). Feedback is provided immediately to indicate whether the response was correct or incorrect. When all trials in the session have been completed, the correct answers are summed up to provide a final score.

The physical board is designed to guide the user on how to lay out the smell vials and how to move and handle them during the session. The user’s desktop together with the printed surface are designed to aid the sorting of not-smelled and already-smelled vials and help guide the interaction.

## Pilot Study

**Figure 4.**
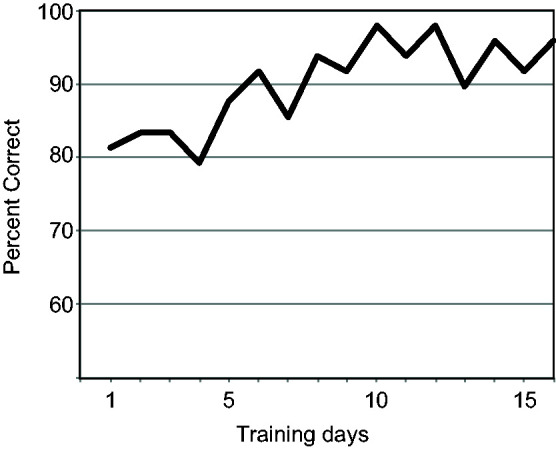
Pilot study; odor training results (percent correct responses over 16 days).

A pilot study was carried out to show a proof of concept; that the method can be used for smell training purposes by repeated assessment over multiple days. One participant (coauthor J. N., a 35-year-old man with no known olfactory dysfunction) provided a daily olfactory self-assessment at home for 16 days. Each day, the participant attempted to identify 48 wine aromas (from the commercially available *Le Nez du Vin* set) using a multiple-choice format with six response options on each trial, five of which were randomly drawn from the odor set and one of which was correct. Corrective feedback was provided on each trial (software is available online at https://osf.io/kwftm/). Through brief training sessions at home (about 10 minutes daily), a gradual improvement from 81% to 96% correct responses was observed over 16 days, offering a proof of principle that the method may be used for olfactory training research (Figure 4). These results should not be interpreted as in any way representative of a population of users, but since no hardware or software errors appeared during this period, this provides an indication that the system is robust and can be used to collect and store olfactory performance data during a multiday training effort.

## User Evaluation Experiment

We conducted a laboratory experiment on naïve users in order to better understand the user experiences on the odor identification task. We recruited 11 adult participants (3 men, 8 women; mean age = 26.6 years, *SD* = 8.36), all reporting normal smell function, and no severe psychiatric or neurologic disorders. Participants were recruited through a designated participant recruitment website, and the research was performed in accordance with the Declaration of Helsinki (noninvasive research of this kind does not require a designated ethical review, according to Swedish law; www.etikprovningsmyndigheten.se). Testing was carried out in a well-ventilated testing room and took approximately 40 minutes. Participants were compensated with a gift card. The assessment was carried out exactly as described in our pilot study, with the addition of a set of questions to assess the user experience at the end of the session. All data were collected for descriptive purposes.

Descriptive results (Table 1) show that the mean odor identification performance accuracy was 66% (*SD* = 14). Performance levels varied across participants (range 38%–85%) but were higher than chance performance (17%) for all participants. The average performance was notably lower than the baseline performance of our pilot participant (who, as noted, had prior knowledge of the odor stimuli and methodology). The performance level of the naïve users indicate that there is ample room for improvement in odor identification training, using our method. We explored the experimental data further, taking advantage of the five odor categories (fruit, floral, vegetal and spice, animal and roasted notes) in the *Le Nez du Vin* set. Descriptive results (Table 1) show that mean accuracy levels were rather similar for the fruit, floral, animal, and roasted notes categories; the vegetal and spice category had a somewhat higher accuracy rate.

**Table 1.** Odor Identification (Percent Accuracy); Overall Score and Scores for the Five Odor Categories.

	Proportion correct	Fruit	Floral	Vegetal and spice	Animal	Roasted
Mean (*SD*)	66 (14)	63 (17)	61 (16)	73 (16)	61 (35)	63 (20)
Median	69	64	60	71	50	57
Range	38–85	29–86	40–83	46–93	0–100	40–100

After completing the assessment, the participants were asked about their impressions of using the Exerscent. Four questions about the system were answered, concerning (1) the overall impression of ease of use, (2) the hardware (reader), (3) the software (computer interface), and (4) the game task. Furthermore, the participants were also asked how much they enjoyed using Exerscent. All responses were provided on a 7-point Likert scale. For the system questions, the scale went from 1 (*very hard*) to 7 (*very easy*, with 4 labeled *neither hard nor easy*). For the question on how fun Exerscent was to use, the scale went from 1 (*not fun at all*) to 7 (*very fun*, with 4 being *neutral*; Table 2). Evaluations of hardware and software questions were very positive, as all participants but one provided the maximum score. The overall enjoyment and ease of use questions yielded high scores, and the task was rated as being of intermediate difficulty. This suggests that the Exerscent system was considered to be very easy and fun to use but that the task was rated to be moderately challenging.

**Table 2.** Perceived Ease of Use and Enjoyment.

How easy was the Exerscent:	Overall	The reader	The software	The game task	How fun was the Exerscent?
Mean (*SD*)	5.64 (1.63)	6.91 (0.30)	6.91 (0.30)	4.55 (0.93)	5.91 (0.94)
Median	6	7	7	5	6
Range	3–7	6–7	6–7	3–6	4–7

*Note*. Answers were provided on a 1- to 7-point scale.

The participants then answered questions on how hard/easy it was to understand (1) the written pretest instructions (how to set up and play the game), (2) the game board, and (3) the instructions on screen. The responses were provided on a scale from 1 (*very hard*) to 7 (*very easy*) and provided ceiling-level results; the instruction sheet was rated as on average 6.73, and the game board and screen instructions were both rated at 7.0 by the participants.

The participants were then asked about their perception of the scents in the game. They were asked how easy it was to *detect* the scents, and how easy it was to identify the scents *with* and *without* the options on the screen. The responses show that on average, it was moderately easy to detect the scents and that odor identification was rather difficult without options provided on the screen but only moderately difficult when options were provided (Table 3).

**Table 3.** Perceived Ease of Olfactory Interaction Aspects.

How easy was it to:	Detect odors?	Identify without options?	Identify with options?
Mean (*SD*)	4.09 (1.22)	2.82 (1.40)	4.45 (1.13)
Median	4	2	5
Range	2–6	1–6	2–6

*Note*. Answers were provided via 1- to 7-point rating scales, with 1 = *very hard*, 4 = *neither hard nor easy*, and 7 = *very easy*.

## Discussion

We have developed an olfactory assessment tool, Exerscent, which fills a gap between olfactory self-assessment methods and the sophisticated olfactometers used in laboratory research (Ischer et al., 2014; Lundström et al., 2010; Niedenthal et al., 2019). In its present design, the device allows for odor identification assessment and game-based training at home. The Exerscent method can be used to provide performance-based assessments of olfaction and how it changes over time, for example, during recovery from postinfectious anosmia or due to other designated training interventions. User evaluations suggest a very positive user experience in a group of participants that varied in terms of task performance levels. Very high ratings on comprehension and enjoyment were obtained even though the odor identification task was rated as moderately difficult (which corresponded with the objective performance level data). These results indicate that the olfactory display meets the goals of good design; to be *efficient*, *easy to learn*, and *enjoyable to use* (Sharp et al., 2007). Collecting trial-by-trial responses enables a number of features that has traditionally been considered beyond reach in olfactory training interventions. For example, analysis of response patterns can be easily automatized and used to adapt the task difficulty along with task performance gains. Notably, our results showed that odor identification scores were quite similar across odor categories, but there was a substantial degree of individual variation (Table 1). Individual error profiles could thus be used to optimize olfactory training protocols. Such methods for individualized olfactory training might be applied in future smell training studies on clinical as well as nonclinical groups.

The Exerscent device might facilitate olfactory research in several ways. First, smell training conducted outside of the lab could be valuable as a therapeutic intervention to counter smell loss, for example, due to coronavirus infection (Gerkin et al., 2020; Hopkins et al., 2020). Here, the Exerscent digital interface will enable more sophisticated data collection and user feedback, and more flexible task designs. Second, the Exerscent might be adapted as a method to expose children to unfamiliar food smells in a game context. Digital game features could thus familiarize children to new food smells, increasing their familiarity without pressure of eating unwanted foods. As familiarity is strongly associated with liking and acceptance, this feature might reduce picky and fussy eating and reduce the risk for long-lasting food aversions (Luisier et al., 2019). Third, Exerscent enables new means of skills acquisition by food, drink, and fragrance professionals who may use Exerscent to cultivate both sensory and cognitive olfactory advantages (Croijmans et al., 2020). Fourth, smell training, especially when adapted to assess both perceptual and cognitive olfactory functions, might be used to provide benefit to aging adults, who often experience joint olfactory and cognitive loss (Josefsson et al., 2017). Apart from these research uses, methods such as the one outlined here may be used for recreational purposes outside of research settings.

Our work highlights the potential of novel technological platforms in conducting olfactory research. Our device is an infrastructure that enables a wide range of applications; of particular interest is olfactory training and research (Gillespie, 2010). However, the broader meanings of the word “platform” suggest a support for activity that is open, egalitarian, and neutral (Gillespie, 2010). This neutrality toward use encourages adoption by a range of users for different purposes, which is a prioritized goal in technological development. We have made our code and instructions freely available online (https://osf.io/kwftm/). In this manner, our device is open for the creation of novel applications. Many forms of interaction can be programmed with our olfactory display hardware, and the quantity and type of scents employed is not determined.

In sum, the Exerscent is a hybrid physical/digital platform that can be used for flexible olfactory assessment and training outside of a laboratory setting. It might create new opportunities for employing digital environments in the service of olfactory research.
